# *In vitro* synthesis and biochemical characterization of acyl-homoserine lactone synthase and its deletion mutant

**DOI:** 10.1371/journal.pone.0304331

**Published:** 2024-05-31

**Authors:** Yechan Jeong, Sunwoo Moon, Jae-hwa Shin

**Affiliations:** Gyeonggi Science High School for the Gifted, Suwon, Gyeonggi, Republic of Korea; Weizmann Institute of Science, ISRAEL

## Abstract

Quorum sensing can induce density-dependent gene expressions that cause various problems. For quorum-sensing inhibition, fundamental solutions such as gene manipulation are required, and acyl-homoserine lactone synthase (AHL synthase), which synthesizes the universal quorum-sensing signal of gram-negative bacteria, can be used as a target. In this study, researchers synthesized His-tagged AHL synthase and its deletion mutant that lacks the active site and compared their biochemical characteristics. His-YpeI, the 6x His-tagged AHL synthase of *Serratia fonticola*, and His-ΔYpeI, its deletion mutant, were designed, and their property conservation were examined using *in silico* projection tools. For *in vitro* synthesis of enzymes, the His-YpeI CFPS template was synthesized by *in vitro* gene synthesis, and the His-ΔYpeI CFPS template was obtained by deletion PCR. CFPS was performed and the products were purified with the 6x His-tag. The enzymes’ properties were compared using an enzymatic assay. The bioinformatic analysis confirmed the conservation of biochemical properties between 6x His-tagged and untagged enzymes, including helix-turn-helix interactions, hydropathy profiles, and tertiary structure between His-YpeI and YpeI and between His-ΔYpeI and ΔYpeI. His-YpeI and His-ΔYpeI synthesized by CFPS were found to have the expected molecular weights and demonstrated distinct differences in enzyme activity. The analyzed enzymatic constants supported a significant decrease in substrate affinity and reaction rate as a result of YpeI’s enzyme active site deletion. This result showed that CFPS could be used for *in vitro* protein synthesis, and quorum sensing could be inhibited at the enzymatic level due to the enzyme active site’s deletion mutation.

## Introduction

Acyl-homoserine lactone (hereafter referred to as AHL) is the most extensively studied gram-negative quorum-sensing signal molecule. The quorum-sensing system mediated by AHL consists of *luxI*/*luxR*-type proteins. *LuxI*-type proteins synthesize AHL, while *luxR*-type proteins receive it [1]. AHL-mediated quorum sensing induces industrially and medically detrimental biofilms and virulence factors. Currently, quorum-sensing inhibitions concentrate on signal analogs or signal degradation, but these techniques are temporal and susceptible to a variety of environmental factors [2].

Consequently, it is essential to investigate methods that manipulate quorum-sensing pathways at the bioengineering level, such as mutating the AHL synthase or the AHL receptor [3, 4]. Former researchers performed gene mutations on the AHL synthase gene in the quorum-sensing pathway and confirmed the efficient inhibition of quorum sensing [5]. Biofilm formation was effectively inhibited through site-directed mutagenesis by deletion PCR.

Cell-free protein synthesis (hereafter referred to as CFPS) is a vital tool in synthetic biology. By using CFPS with only the template and cell lysate, the desired protein can be easily obtained within a short period *in vitro* [6, 7]. The CFPS template consists of three regions: the target gene, promoter, and terminator upstream and downstream of the target gene. The promoter and terminator must be appropriate to the cell lysate for successful *in vitro* transcription. The basic principle of CFPS is to use cell lysate, a designated master mix, and cell extract to transcript mRNA and translate it from the CFPS template *in vitro*. Furthermore, the codon usage of cell lysate, known as codon bias, must be considered for high translation efficiency [8]. By performing CFPS at the appropriate temperature, we can obtain the crude protein mixture containing some expression proteins and the desired protein.

Polyhistidine tags are currently the most widely-used method in protein purification by affinity. By using immobilized metal ions in the resin and protein with a polyhistidine tag, the desired protein could easily be bound to the resin. Polyhistidine tags are known to bind to divalent metal cations (e.g., Mn^2+^, Co^2+^, Ni^2+^) and be eluted by buffer with imidazole, which can also be competitively bound to divalent metal cations [9]. Since the 6x His-tag is small, easy to add to the N/C-terminus, and less disruptive to the protein property or structure, the 6x His-tag is one of the most-used tags in protein purification [10]. However, validating the functional preservation of 6x His-tagged proteins must still be performed via various molecular biology tools.

In this study, enzymatic assays of 6x His-tagged AHL synthase and its deletion mutant obtained by CFPS were performed, while a bioinformatic study was used to validate the enzymatic assay. Through this process, the biochemical characteristics of the 6x His-tagged AHL synthase were shown to differ from its deletion mutant. This result demonstrated that CFPS and the enzymatic assay are effective tools for validating the characteristic of enzymes while using the bioinformatics tool. Additionally, results validate that inhibition of quorum sensing using deletion mutation is an effective strategy at the biochemical level.

## Materials and methods

### *In silico* projection of proteins’ properties

The *Serratia fonticola*’s *luxI*-type gene, *YpeI* (Gene ID: 30320740), which synthesizes AHL, was the gene of interest. The total DNA sequence of the gene was determined, and the amino acid sequence corresponding to the DNA sequence was confirmed, referred to by previous research [11]. From the DNA sequence of the expression vector pBT7-N-His, the sequence of the linker and the 6x His-tag was determined and added to the upstream of *YpeI*. This new gene, which encodes wild-type AHL synthase of *S*. *fonticola* with a 6x His-tag at the N-terminus, was named *His-YpeI*. The DNA region encoding the enzyme active site of YpeI, as determined by previous research, was removed from *YpeI* to obtain *ΔYpeI*, which encodes the enzyme-inactive deletion mutant of YpeI [5]. To determine *His-ΔYpeI*, which encodes the deletion mutant of His-*ΔYpeI* lacking the enzyme active site, the identical enzyme active site coding region was deleted.

To validate that 6x His-tag does not change the biochemical property of the enzyme, structural conservation, and the biochemical level comparison of each protein; the following steps were conducted. Structural prediction of the proteins was preceded by predicting the biochemical properties of four proteins encoded by these genes by running AlphaFold version 2, which was used to project the tertiary structure of each protein. The primary structures of the proteins, obtained by translating each gene, were used for the input data. To ensure the feasibility of the structure, the predicted local distance difference test (from now on referred to as pLDDT) was measured. pLDDT is the per-residue accuracy of the structure, corresponding to the model’s predicted score on the IDDT-Cα [12]. For the comparative structure analysis of these proteins, the protein structure comparison computation program Flexible structure AlignmenT by Chaining Aligned fragment pairs allowing Twists (FATCAT) was executed with the tertiary structures of the proteins obtained from AlphaFold version 2 [13]. Pairwise alignment and structure comparisons were conducted. In addition, the 3-dimensional orientation of the 6x His-tag was checked to avoid His-tag enzyme malfunction.

A hydropathy plot was figured out and compared to understand the hydropathic properties of the proteins. Kyte & Doolittle hydropathy index was used, and the entire sequence of each protein was plotted [14]. The window size was set to 13 amino acids, the average strand size, and the helices size of the protein. A linear weight variation model was used, and the weight of the edge was set as 10% of the weight of the window center. The normalization of the index was not performed.

Characteristic forms of each protein were explored to ensure the conservation of the biochemical properties during the addition of a 6x His-tag and linker sequence. The helix-turn-helix (HTH) regions of four proteins were predicted using Network Protein Structure Analysis (NPS@) [15]; identical amino acid sequence pairs of the proteins were observed. To examine the electrostatic interaction of the residue in HTH regions, the helical wheel and projection net were analyzed. The program, NetWheels, was used to obtain data, and the first/last ratio for the analysis was standardized to 1. Each helical wheel and net projection were analyzed solely with the HTH region of the protein.

### *In vitro* synthesis of His-YpeI and His-ΔYpeI

**Fig 1:**
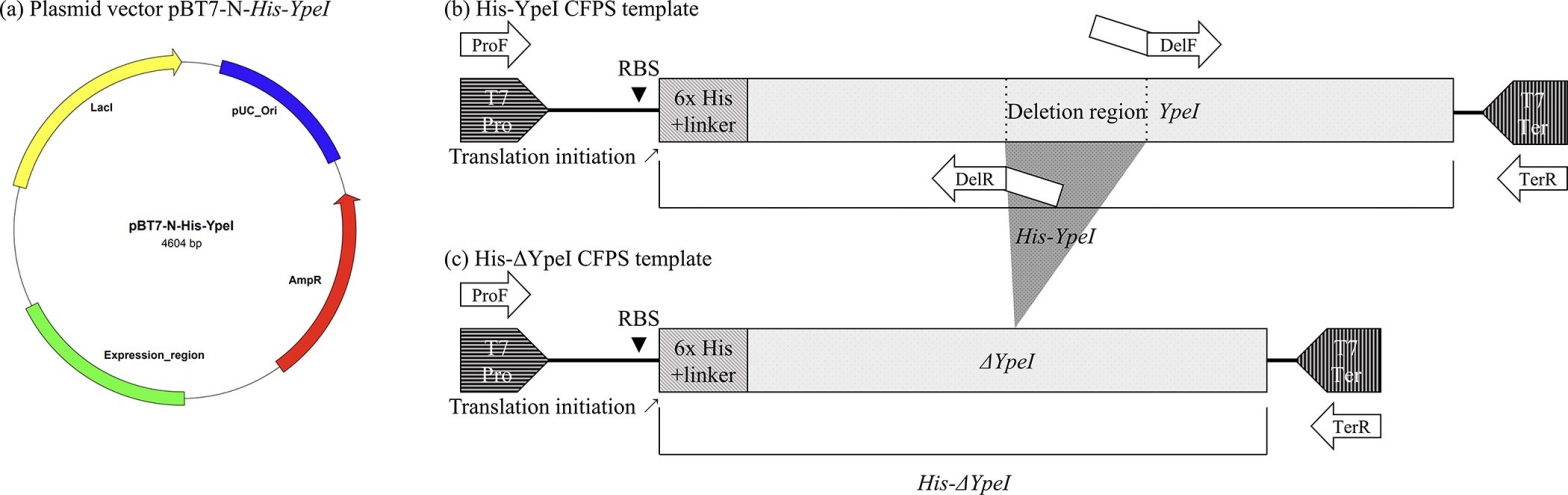
CFPS template synthesis. (a) The structure of pBT7-N-His-YpeI (b) Structure and primers to obtain the His-YpeI CFPS template (c) Structure and primers to obtain the His-ΔYpeI CFPS template.

Before synthesizing the His-YpeI CFPS template, *YpeI* was cloned to the expression vector pBT7-N-His (Fig 1(A)). Upstream of the cloning region of the vector, pBT7-N-His contains 6x His-tag and linker sequence, which allows the formation of *His-YpeI* by cloning to pBT7-N-His. At the upstream of this gene, the T7 promoter sequence and the DNA sequence for the ribosomal binding site are located. At the downstream of the gene, the T7 terminator sequence is located. In the region between the T7 promoter and the T7 terminator (expression region), *His-YpeI* could be expressed. To obtain the recombinant vector pBT7-N-*His-YpeI*, which has *YpeI* at the cloning site of pBT7-N-His, gene synthesis and cloning services were used (Gene Synthesis, Bioneer, South Korea). Gene synthesis was performed via DNA oligomer synthesis and assembly *in vitro*. Since CFPS only requires the expression region containing the gene, PCR was performed with ProF, TerR primer set, using pBT7-N-*His-YpeI* as the template. ProF is the primer that binds to the T7 promoter, and TerR is the primer that binds to the T7 terminator. This result made the His-YpeI CFPS template (Fig 1(B)).

To obtain the His-ΔYpeI CFPS template, deletion PCR was performed (Fig 1(C)). Deletion PCR and its primer principles are as follows; it comprises two stages. The first PCR involves the amplification of the regions upstream and downstream of the deletion region. Thus, two sets of primers are employed for the synthesis of two fragments from the template incorporating complementary sequences from each product. In the second PCR, the two fragments obtained from the first PCR are spliced together by overlapping the complementary sequences. The second PCR employs the reverse primer located upstream and the forward primer located downstream of the deletion region [5]. The region upstream of the enzyme active site binds to the reverse primer named DelR. Similarly, the region downstream of the enzyme active site binds to the forward primer, named DelF. These two primers possess both an annealing region and a nonannealing region and are intended to have an overlapping sequence of the nonannealing region. The annealing regions of ProF, TerR, DelF, and DelR primers are designed to have a difference within 2°C in T_m_ value (Table 1). The first PCR was performed with the ProF, DelR primer set, and DelF, TerR primer set using the His-YpeI CFPS template as the template. The PCR products were electrophoresed and gel-extracted to solely contain PCR products and exclude template DNA (TaKaRa MiniBEST Agarose Gel DNA Extraction Kit, TaKaRa, Japan). The second PCR was performed with the ProF, TerR primer set, which acquired the two DNA fragments from the first PCR in the same mass ratio with the length ratio (the concentration contribution). All PCRs used the HotStart application with pfu polymerase for the high accuracy of PCR (AccuPower^®^ HotStart PCR PreMix, Bioneer, South Korea). Through this process, the His-ΔYpeI CFPS template was synthesized. The confirmation of successful deletion was confirmed by 1.5% agarose gel electrophoresis.

**Table 1:** PCR primers.

Name	Sequence (5’ to 3’, annealing region)	Purpose (F/R)
ProF	TAATACGACTCACTATAGGGGAA	CFPS template (F)
TerR	GCTAGTTATTGCTCAGCGG	CFPS template (R)
DelF	GCGTGGAGAACGGCATGTCACTGATTAACTATGCGCA	Deletion PCR (F)
DelR	GCATAGTTAATCAGTGACATGCCGTTCTCCACG	Deletion PCR (R)

To obtain His-YpeI and His-ΔYpeI, CFPS was performed. The T7 promoter and terminator were used for the expression of the gene. PCR purification was performed with each PCR product to obtain CFPS templates solely (QIAquick PCR Purification Kit, Qiagen, USA). Next, using 600 ng of DNA as a template with an *E*. *coli* extract expression system, the mixture was made according to the manual and incubated at 30°C for 3 h (AccuRapid™ Protein Expression Kit, Bioneer, South Korea). Purification was performed since the product contains other proteins from *E*. *coli* extract. TALON^®^ resin (with immobilized Co^2+^ ion) was used for affinity purification of 6x His-tagged proteins [16]. The magnetic bead bound with TALON^®^ resin was used to purify His-YpeI and His-ΔYpeI from the crude protein mixture (TALON^®^ Magnetic Beads, TaKaRa, Japan). Next, 200μL of magnetic beads were used, following the manual protocols. SDS-PAGE of crude reactants, non-absorbed materials, and purified products was performed using 15% polyacrylamide gel to validate protein purification for a single type of protein (6x His-tagged). After SDS-PAGE, the gel was wet-transferred to the PVDF membrane for higher sensitivity, the protein bands were stained with CBB-R-250, and the signals of obtained bands were quantified to ensure that the eluted protein solely contained 6x His-tagged proteins (Mini-PROTEAN^®^ Tetra Cell and Mini Trans-Blot^®^ Module, Bio-Rad, USA).

### Biochemical characterization of His-YpeI and His-ΔYpeI

**Fig 2:**
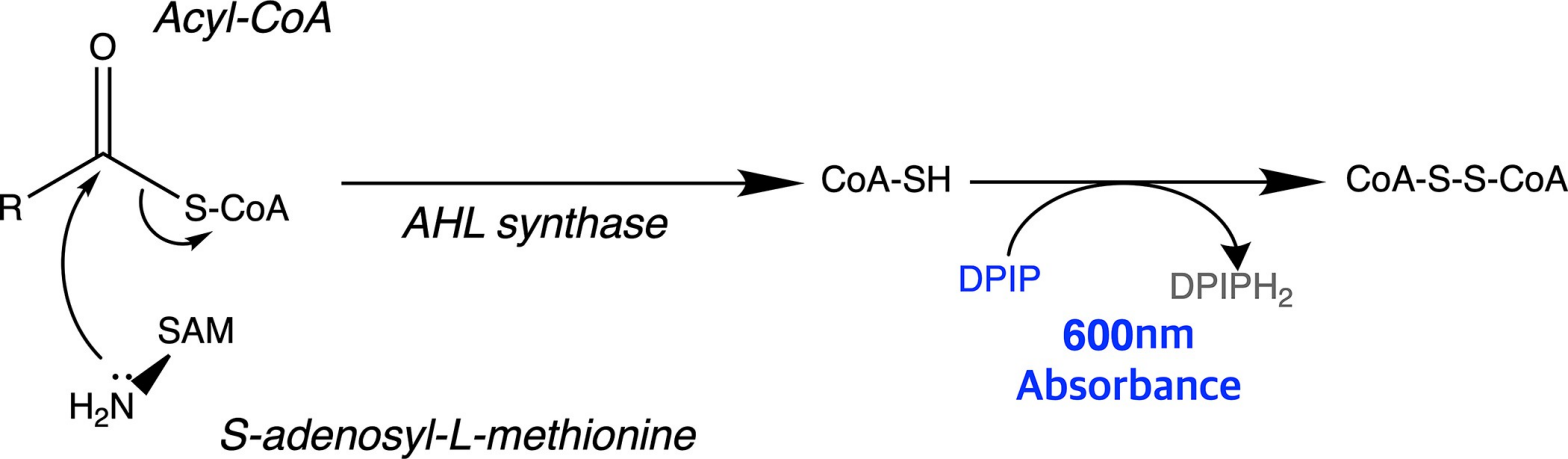
Enzymatic assay using DPIP reduction. AHL synthesis and DPIP reduction to quantify enzyme activity.

The enzymatic assay was performed to examine the properties of His-YpeI and His-ΔYpeI to examine the effect of deletion mutation. AHL synthase uses acyl-acyl carrier protein (acyl-ACP) or acyl-coenzyme A (acyl-CoA) and S-adenosyl-L-methionine (SAM) as substrate [17]. In the presence of AHL synthase, nucleophilic attack of the SAM α-amino group to the C-1 carbonyl carbon of the acyl-ACP or acyl-CoA occurs (acylation), producing holo-ACP or HS-CoA from acyl-ACP or acyl-CoA, respectively [18]. Subsequently, a nucleophilic attack of α-carboxylate oxygen on the γ-carbon of SAM occurs, which is lactonization of SAM [18]. This results in the production of AHL and 5’-methyl-thioadenosine. An AHL synthase enzymatic assay could be conducted by quantifying one of these products. In this study, we used the quantification of HS-CoA. In the presence of 2,6-Dichlorophenolindophenol (DPIP), a redox reaction occurs. HS-CoA is oxidized and reduces DPIP to DPIPH_2_ [19]. The concentration of DPIP can be monitored by a colorimetric absorbance of 600 nm in real-time. This can quantify the rate of HS-CoA released by the enzyme activity of AHL synthase (Fig 2).

The stock solutions of DPIP (2,6-Dichloroindophenol sodium salt hydrate, Sigma–Aldrich, USA), SAM (S-(5′-Adenosyl)-L-methionine iodide, Sigma–Aldrich, USA), and hexanoyl-CoA (Hexanoyl-coenzyme A lithium salt hydrate, Sigma–Aldrich, USA) were prepared in ultrapure water. The stock solution of 1M HEPES buffer (pH 7.3) (1M HEPES, Enzynomics, South Korea) was also prepared. Before the enzymatic assay, quantification of the eluted protein was performed. The BCA assay (Pierce™ BCA Protein Assay Kit, Thermo Fisher Scientific, USA) was performed with bovine serum albumin standard for the quantification. After quantification, the protein was diluted to the optimal concentration using the elution buffer mixture of the purification kit and ultrapure water.

The protocol of the AHL synthase assay is applied from the general method using the DPIP redox reaction, with a specification to YpeI [18]. The protocol consists of three steps: determining background rates, determining optimal enzyme concentration, and specifying the enzymatic constant.

The determination of background rates was conducted through the following process. All components except the AHL synthase enzyme were added to the microplate well. The enzyme-free progress curve was recorded at 600 nm for 15–20 minutes. The minimum incubation time required to reduce nonspecific background reactions in the enzymatic assay was determined by measuring the time required for the absorbance change to stabilize. The background rate was determined by monitoring the progress curve for an additional 5 min and calculating its slope. The experiment was repeated with hexanoyl-CoA as the fixed substrate to determine the background rate in the enzymatic assay for each concentration variation.

To determine the optimal enzyme concentration, the initial rates for four different enzyme concentrations from 0.1–2 μM were determined. The reaction rate at the lowest hexanoyl-CoA concentration was selected three-fold higher than the background rate.

Except for the enzyme, the reaction mixture was primarily incubated for the minimum incubation time. After the enzyme was added, the absorbance at 600 nm was collected for 5 min, and the linear slope between 30–200 sec was calculated. The initial rate of each enzyme reaction, ΔAbsorbance/sec, was converted to μM/min. Then, the background rate was subtracted from this rate to calculate the net enzymatic rate, and the assay was repeated for each substrate concentration in triplicate. The initial rate vs. substrate concentration data were fitted to the Michaelis–Menten model using nonlinear regression, and kinetic constants were determined. The differences between enzymes and significance were tested using the student’s t-test.

## Results and discussion

### *In silico* projection of proteins’ properties

**Fig 3:**
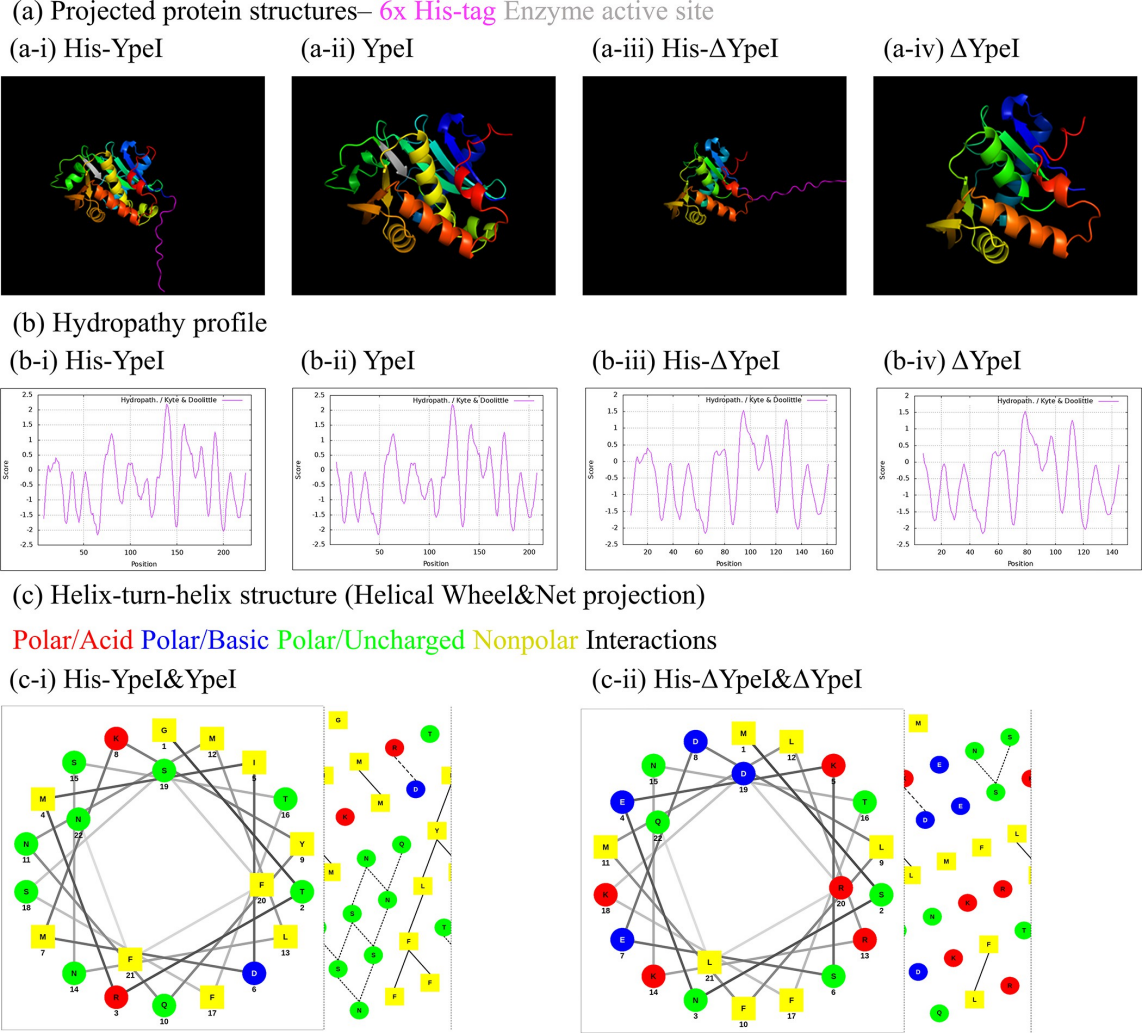
*In silico* projections. (a) Projected tertiary structures of (a-i) His-YpeI, (a-ii) YpeI, (a-iii) His-ΔYpeI, (a-iv) ΔYpeI (b) Hydropathy plots of (b-i) His-YpeI, (b-ii) YpeI, (b-iii) His-ΔYpeI, (b-iv) ΔYpeI (c) Helical wheels and net projection diagrams of the projected HTH regions of (c-i) His-YpeI and YpeI, (c-ii) His-ΔYpeI and ΔYpeI.

The biochemical and structural properties of YpeI, ΔYpeI, His-YpeI, and His-ΔYpeI were predicted. The proteins’ tertiary structures were projected by running AlphaFold version 2. Thus, to ensure the feasibility of structure prediction, pLDDT was measured. The pLDDT of the predicted protein structure of YpeI, ΔYpeI, His-YpeI, and His-ΔYpeI were 93.6, 87, 89.7, and 80.8, respectively. pLDDT over 70 are expected to be well-modeled, which supports the precise structure prediction. FATCAT was run with the tertiary structures of the proteins obtained from AlphaFold version 2 for comparative structure analysis. The YpeI and His-YpeI set has a p-value of 0, and ΔYpeI and His-ΔYpeI set has a p-value of 0. The YpeI and ΔYpeI set has a p-value of 2.42E-12, and the His-YpeI and His-ΔYpeI set has a p-value of 2.73E-11. Therefore, any two of YpeI, ΔYpeI, His-YpeI, and His-ΔYpeI, form a pair with a p-value of <0.01, meaning they are significantly similar in structure. Furthermore, the 3-dimensional orientation of the 6x His-tag was checked to ensure the 6x His-tagged enzyme’s function conservation (Fig 3(A)).

To understand the hydropathic properties of the proteins, a hydropathy plot using Kyte & Doolittle hydropathy index was obtained and compared using the Kyte & Doolittle hydropathy index. YpeI and His-YpeI resulted in a similar plot, only differing in the N-terminus part where 6x His-tag and linker sequence were added. Proteins ΔYpeI and His-ΔYpeI also showed a similar result. However, YpeI and ΔYpeI showed a difference in most parts of the plot, resulting from the active site’s deletion. This shows how the addition of the 6x His-tag and linker sequence does not change the hydropathic properties of the proteins. Furthermore, this result supports that the deletion mutation of the enzyme active site changes the overall hydropathic property of the enzyme while its structure is conserved (Fig 3(B)).

The HTH regions of four proteins were predicted using NPS@ to ensure the conservation of the biochemical properties during the addition of the 6x His-tag and linker sequence. YpeI and His-YpeI showed the same result with GTRMIDMKYQNMLNSTFSSFFN as the HTH sequence. ΔYpeI and His-ΔYpeI, with MSNEKSEDLFMLRKNTFKDRLQ as the HTH sequence, also showed the same result. However, YpeI and ΔYpeI showed a difference in most parts of the result, which is induced by the deletion of the active site. HTH regions are characteristic structures of a protein; thus, the results of the analysis of the HTH regions can show that the addition of the 6x His-tag and linker sequence doesn’t change the properties of the proteins. To further examine the change in amino acid residue interaction in the HTH regions, we obtained a helical wheel and net projection diagram on the projected HTH sequence. The helices’ different transversal and coronal interactions were observed between His-YpeI and YpeI set to the His-ΔYpeI and ΔYpeI. This supports the idea that mutation leads to the biochemical level change in the enzyme, although the overall enzyme structure is conserved (Fig 3(C)).

### *In vitro* synthesis of His-YpeI and His-ΔYpeI

**Fig 4:**
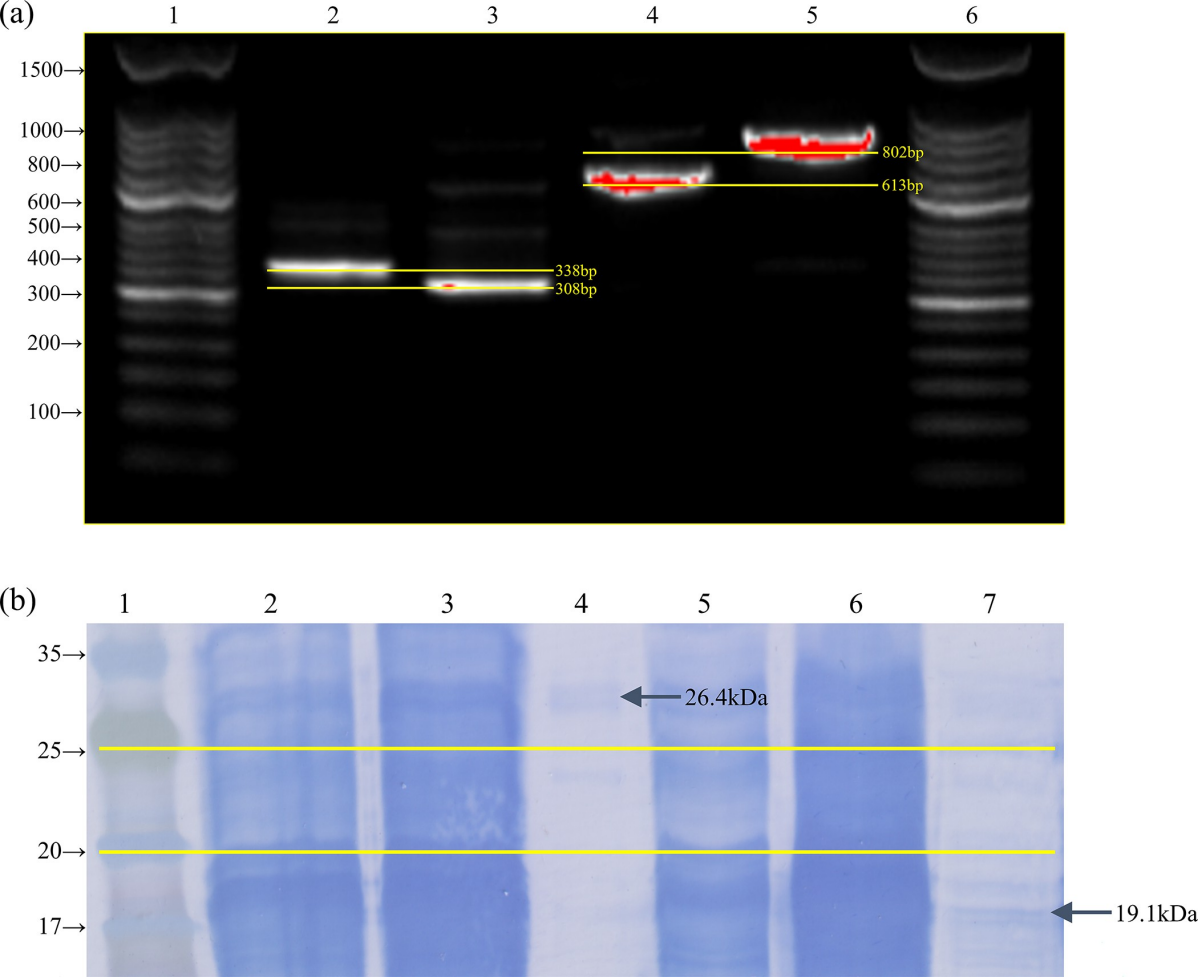
*In vitro* synthesis of enzymes. (a) DNA electrophoresis results to confirm the CFPS templates (b) Transferred SDS-PAGE result to confirm the synthesis and purification of the proteins.

To obtain the proteins His-YpeI and His-ΔYpeI, CFPS templates were synthesized for each protein while electrophoresis was performed to confirm the synthesis of CFPS templates. Lanes 1 and 6 are the DNA size marker, lane 2 is fragment 1 to synthesize the His-ΔYpeI CFPS template, which is 338 bp, and lane 4 is fragment 2 for synthesizing the His-YpeI CFPS template, which is 308 bp. The final CFPS templates are in lanes 4 and 5. Lane 4 is the His-YpeI CFPS template, 613 bp, and lane 5 is the His-YpeI CFPS template, 802 bp (Fig 4(A)).

CFPS reactions were performed with purified PCR products of both templates. After incubation, the crude reactants were purified. Purified products (final eluents), nonabsorbed materials, and crude reactants were analyzed by SDS-PAGE and its transferred result. Lane 1 is the protein size marker, lanes 2–4 are about His-YpeI, and 5–7 are about His-ΔYpeI. Lanes 2 and 5 are crude reactants, lanes 3 and 6 are nonabsorbed materials, and lanes 4 and 7 are purified products. The band of each purified product supports the successful purification of each protein. Additionally, the protein molecular weights, 26.4 kDa of His-YpeI and 19.1 kDa of His-ΔYpeI, were confirmed, due to the deletion mutation. Lane 4’s purified product, with a molecular weight of approximately 26.4kDa, corresponds to the intended product synthesized through CFPS. Lane 7’s purified product, with a molecular weight of around 19.1kDa, also aligns with this. The absence of additional bands in each lane indicates the specific purification and successful synthesis of the CFPS product (Fig 4(B)).

### Biochemical characterization of His-YpeI and His-ΔYpeI

**Fig 5:**
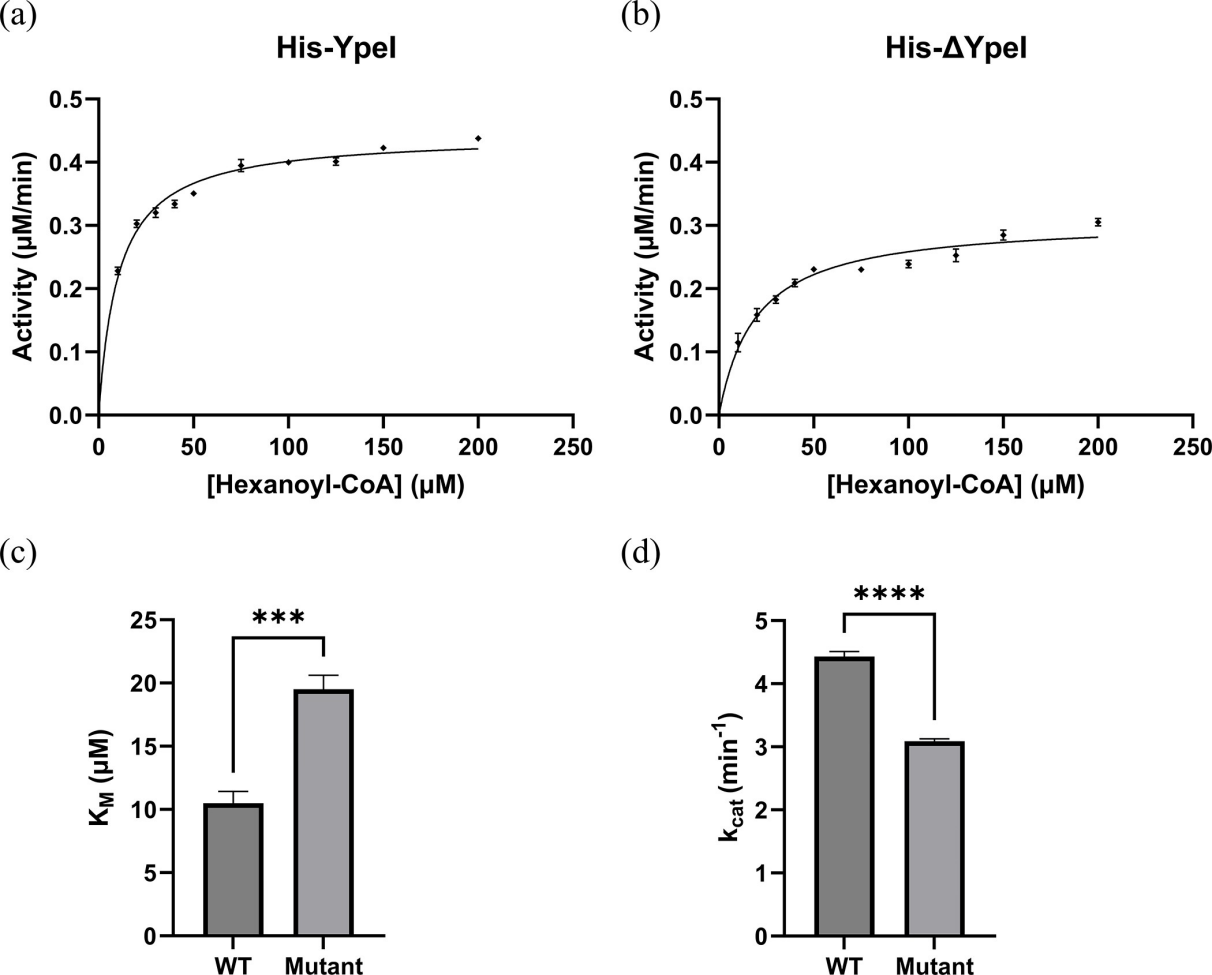
Enzymatic assay results. (a) Michaelis-Menten curve of His-YpeI (b) Michaelis-Menten curve of His-ΔYpeI (c) Comparison of the *K*_*M*_, means and standard errors are indicated (*** denotes p<0.0005) (d) Comparison of the *k*_*cat*_, means and standard errors are indicated (**** denotes p<0.0001).

The optimal condition for the enzymatic assay was determined as the following conditions. The optimal concentration for each enzyme was 0.1 μM under 400 μM of SAM. The 10 hexanoyl-CoA concentrations are varied between 10 and 200 μM. Under optimal conditions, the following data were obtained and fitted into the Michaelis–Menten model (Fig 5(A) and 5(B)). Based on the obtained data and initial enzyme concentration, the Michaelis–Menten constants (*K*_*M*_) of the enzymes and turnover rates (*k*_*cat*_) were determined, and both enzymatic constants showed significant differences. In a 95% confidence interval, *K*_*M*_ increased on the His-ΔYpeI from His-YpeI’s 10.47 ± 1.39 μM to 19.48 ± 4.24 μM, and based on the t-test for the obtained *K*_*M*_ set, the significance of the difference showed p < 0.0005 (Fig 5(C)). In a 95% confidence interval, the *k*_*cat*_ decreased on the His-ΔYpeI from His-YpeI’s 4.433 ± 0.104 min^−1^ to 3.088 ± 0.159 min^−1^, and based on the t-test for the obtained *k*_*cat*_ set, the significance of the difference showed p < 0.0001 (Fig 5(D)). A higher *K*_*M*_ value of His-ΔYpeI to His-YpeI denotes the decrease in substrate affinity, and a decrease in the *k*_*cat*_ value of His-ΔYpeI shows the decrease in the overall reaction rate due to the lack of the enzyme active site. Although the His-ΔYpeI’s enzyme active site was deleted, it appears that some of the reactions proceeded due to the background reaction and other structural catalysis of the enzyme [19, 20].

These results indicate that quorum sensing could be effectively inhibited at the molecular and biochemical levels by the deletion mutation on the enzyme active site coding region of the *luxI*-type gene, and they suggest *in vitro* validation of enzyme activity using only an *in vitro* system and bioinformatics tools throughout the research. The experimental design of this study, solely using *in vitro* systems, validated the rapid and precise characterization of proteins without an *in vivo* system. Further research may suggest CFPS to be widely utilized as the tool for *in vitro* synthesis of proteins. We expect this discovery to lead to the establishment of the method for rapid screening of enzymes solely using *in vitro* systems.

## Supporting information

S1 Dataset (XLSX)

S1 File Protein model of His-YpeI. AlphaFold result of His-YpeI tertiary structure projection.
(PDB)

S2 File Protein model of YpeI. AlphaFold result of YpeI tertiary structure projection.
(PDB)

S3 File Protein model of His-ΔYpeI. AlphaFold result of His-ΔYpeI tertiary structure projection.
(PDB)

S4 File Protein model of ΔYpeI. AlphaFold result of ΔYpeI tertiary structure projection.
(PDB)

S1 Fig DNA electrophoresis result. (TIF)

S2 Fig Transferred SDS-PAGE result. (TIFF)
