# Supplementary figures and images for: In vitro synthesis and biochemical characterization of acyl-homoserine lactone synthase and its deletion mutant
Source: PLoS One. 2024 May 31;19(5):e0304331. doi: 10.1371/journal.pone.0304331 (PMC11142500; doi:10.1371/journal.pone.0304331)

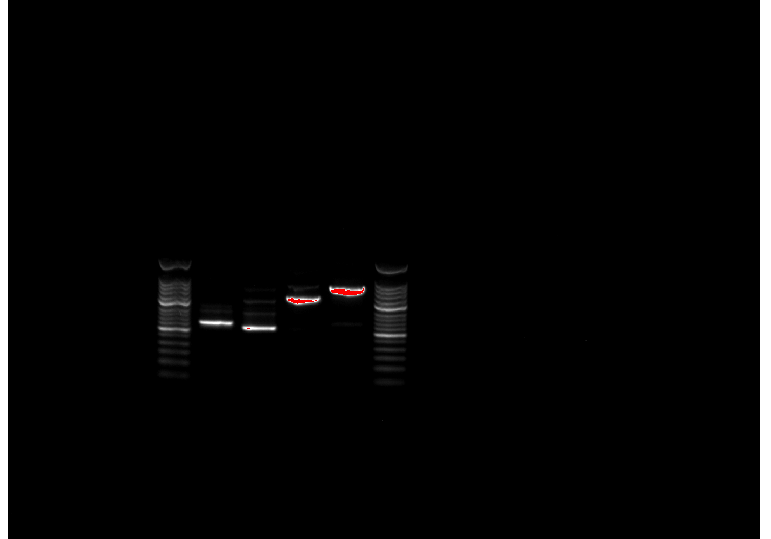

Supplement: S1 Fig — (TIF) [file pone.0304331.s006.tif]

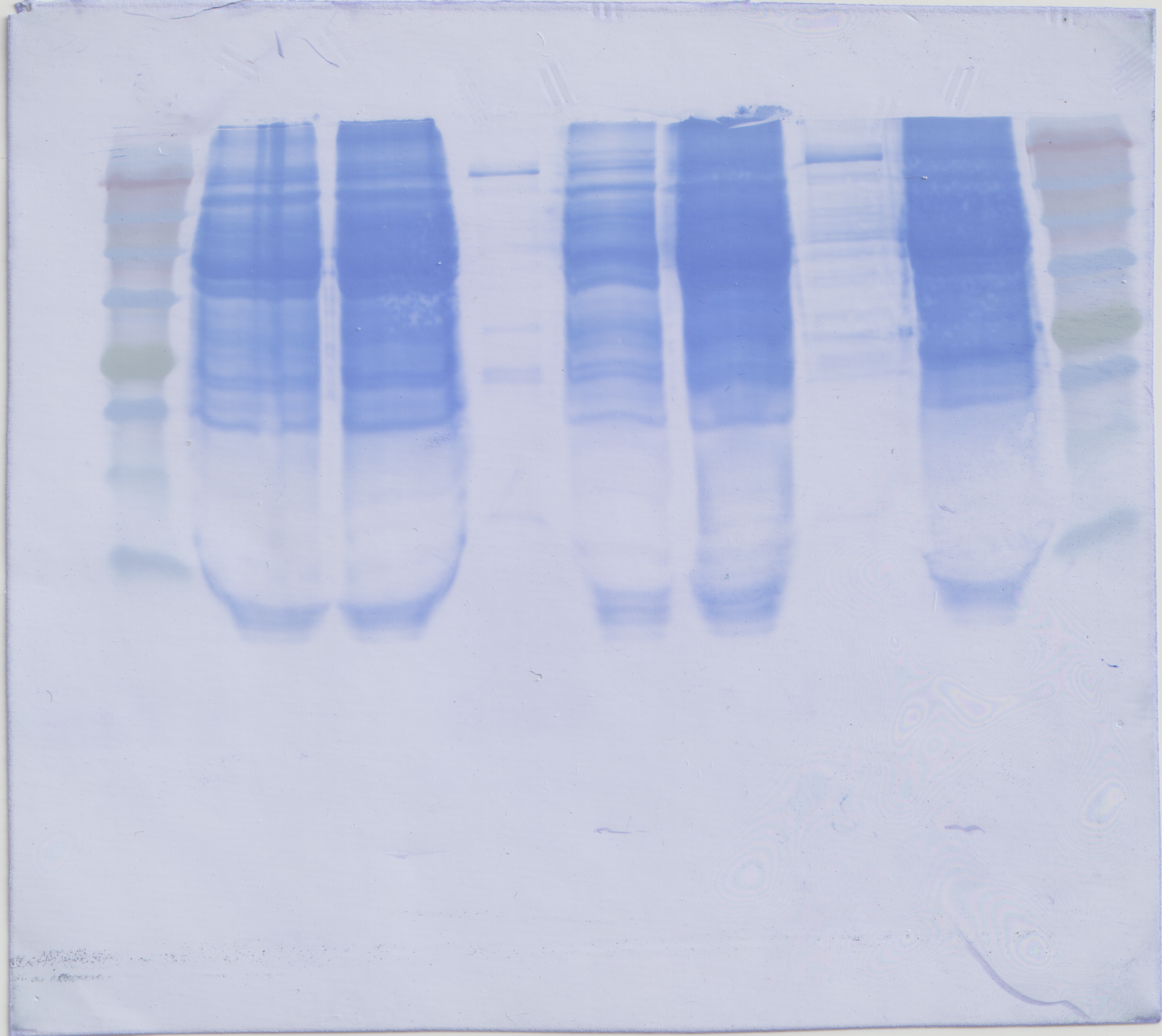

Supplement: S2 Fig — (TIFF) [file pone.0304331.s007.tiff]
